# Exogenous Enzymes Influenced *Eimeria*-Induced Changes in Cecal Fermentation Profile and Gene Expression of Nutrient Transporters in Broiler Chickens

**DOI:** 10.3390/ani11092698

**Published:** 2021-09-15

**Authors:** Yang Lin, Oluyinka A. Olukosi

**Affiliations:** Department of Poultry Science, University of Georgia, Athens, GA 30602, USA; Yang.Lin@uga.edu

**Keywords:** xylanase, protease, *Eimeria*, short-chain fatty acid, cecal fermentation, tight junctions, nutrient transporter, broiler chicken

## Abstract

**Simple Summary:**

*Eimeria*-induced coccidiosis, a common disease in the poultry industry, causes substantial economic loss globally. The developed resistance to synthetic anticoccidial drugs and increasing public and legislative pressures to decrease antibiotic utilization drive research exploring non-antibiotic alternatives to control coccidiosis. Two experiments were conducted to investigate the potential mechanisms by which enzymes may mitigate the negative effects of *Eimeria* on growth performance, nutrient-transporter gene expression, and cecal fermentation patterns. The results demonstrated that *Eimeria* changed the expression of tight junctions and nutrient transporters genes, promoted cecal protein fermentation, and inhibited cecal saccharolytic fermentation. Exogenous xylanase and protease supplementation alleviated negative effects of *Eimeria* effects on the above responses, and thus demonstrated benefits of enzyme supplementation beyond improvement in nutrient utilization.

**Abstract:**

Two 21-day experiments were conducted to investigate the effects of exogenous enzymes on growth performance, tight junctions, and nutrient transporters, jejunal oligosaccharides and cecal short-chain fatty acids (SCFA) of broiler chickens challenged with mixed *Eimeria*. Two different basal diets: high fiber-adequate protein (HFAP; Expt. 1) or low fiber-low protein (LFLP; Expt. 2) were used in the two experiments. In each experiment, birds were allocated to four treatments in a 2 × 2 factorial arrangement (with or without protease and xylanase combination; with or without *Eimeria* challenge). In Expt. 1, with HFAP diets, *Eimeria* upregulated (*p* < 0.05) the expression of claudin-1, but downregulated (*p* < 0.05) glucose transporters GLUT2/GLUT5. On the contrary, enzymes downregulated (*p* < 0.05) claudin-1 and alleviated the *Eimeria*-depressed GLUT2/GLUT5 expression. In both experiments, *Eimeria* decreased (*p* < 0.05) cecal saccharolytic SCFA and increased (*p* < 0.05) cecal branched-chain fatty acids. The challenge × enzyme interaction (*p* < 0.05) showed that enzymes reversed the *Eimeria* effects on fermentation pattern shift. In conclusion, *Eimeria* altered tight junctions and nutrient transporters expression promoted cecal proteolytic fermentation and inhibited saccharolytic fermentation. Exogenous enzymes showed the potential of alleviating the *Eimeria*-induced intestinal gene expression changes and reversing the unfavorable cecal fermentation pattern.

## 1. Introduction

Avian coccidiosis is a worldwide disease that is caused by protozoan parasites of *Eimeria* spp. Seven species of *Eimeria* including *E. acervuline*, *E. brunetti*, *E. maxima*, *E. mitis*, *E necatrix*, *E. praecox*, and *E. tenella* have been identified in the broiler chicken industry [1]. The prevalence of coccidiosis usually occurs with a mixed infection instead of a single species with the mixed infection by *E. acervulina*, *E. maxima*, and *E. tenella* being more prevalent [2]. These three species of *Eimeria* spp. preferentially invade and multiply in the regions of duodenum, jejunum plus ileum, and ceca, respectively; however, intersectional infection also can be observed [3]. 

Damage to gastrointestinal epithelial cells resulting in depressed nutrient utilization and growth performance in birds leads to USD 14.5 billion losses annually in poultry production due to coccidiosis [4,5]. *Eimeria*-induced gastrointestinal damage is also considered to influence the regulation of intestinal tight junctions and nutrient transporters. The changes to intestinal nutrient transporters including sugars, amino acids, and mineral transporters may partly contribute to the *Eimeria*-induced growth depression [6,7]. In addition, it has been demonstrated that *Eimeria* infection impacted the microbial population and composition in the hindgut of broiler chickens, resulting in cecal dysbacteriosis. The infection decreased the abundance of beneficial bacteria such as carbohydrate-fermenting *Ruminococcaceae*, butyrate-producing *Faecalibacterium*, and probiotic bacterium *Lactobacillus*, whereas enriched opportunistic pathogenic bacteria of the genera *Enterococcus* and *Streptococcus* [8]. The dysbacteriosis in the hindgut indicates a shift of fermentation patterns in ceca.

Anticoccidial drugs and live vaccines have been applied in the commercial poultry industry as conventional methods to prevent coccidiosis. However, after exposure to antibiotics for decades, *Eimeria* spp. have developed resistance to these synthetic anticoccidial drugs whereas vaccines are costly for production and storage [9]. In view of the need for alternatives to antibiotics, dietary antibiotic alternatives are possible options to help treat or prevent coccidiosis. Probiotics such as *Lactobacillus*-based probiotic, *Pediococcus acidilactici*-based probiotic, and *B. subtilis*-based probiotics have shown positive results in enhancing mucosal immunity, lessening *Eimeria*-induced growth depression, and improving resistance to *Eimeria* infection [10,11,12]. Prebiotics with the ability to maintain intestinal flora balance has also shown promise by enriching beneficial bacteria [12]. 

It has been reported that the effects of prebiotic and carbohydrase supplementation on the performance of broilers challenged with coccidia are similar, supporting the hypothesis that oligosaccharides are released from carbohydrase-degraded non-starch polysaccharides and play prebiotic roles in birds’ gastrointestinal tract [13,14,15]. In a previous study with supplementation of protease alone or in combination with carbohydrases, the effects of enzymes on the disappearance of non-starch polysaccharides in digesta and accumulation of lower molecular weight carbohydrates were similar [16]. In view of the potential effects of enzyme supplementation and dietary fiber profile affecting the generation of prebiotic substances in the digestive tract of birds [17], there is a possibility of these factors having positive effects on the intestinal milieu of broiler chickens challenged with coccidiosis. Consequently, two experiments were conducted to investigate the potential and mechanism of enzymes on mitigating the negative effects of *Eimeria* challenge on growth performance, gene expression of nutrient transporters, and cecal fermentation patterns. A two-diet model, high fiber-adequate protein, and low fiber-low protein were chosen from previous work [17], where xylanase and protease showed effects on digesta concentration of oligosaccharides and cecal short-chain fatty acids (SCFA). Consequently, these diets were used separately in the current experiment because their interactions in a factorial treatment arrangement setting have been demonstrated previously [17]. 

## 2. Materials and Methods

### 2.1. Animals, Diets Experimental Design and Eimeria Challenge

One hundred and twenty zero-day-old Cobb 500 male broiler chicks were used in each of the two 21 d experiments. A basal diet, formulated to have a high level of fiber and adequate protein (HFAP), was used in Expt. 1, whereas the basal diet in Expt. 2 was formulated to have a low level of fiber and lower than recommended protein level (LFLP). Each of the experiments had four treatments in a 2 × 2 factorial arrangement. The factors were enzyme supplementation (with or without a combination of xylanase and protease) and *Eimeria* challenge (with or without). The birds in all the treatments in each experiment had the same initial body weight (day 0). Each of the four treatments had six replicate cages and five birds per replicate cage. Light was provided for 24 h on the first three days and gradually decreased to 8 h dark period on the last day. The room temperature was set at 33 °C and gradually decreased to 25 °C during the rearing period. Temperature and humidity were recorded daily.

The basal diets were corn-soybean meal-based, and wheat plus wheat bran was used as a fiber provider in the HFAP diet, producing a slightly lower AME level in Expt. 1 diet. The low-protein diets used in Expt. 2 had three percentage points lower crude protein levels compared with adequate protein diets. Five hundred FTU/kg phytase (Quantum Blue, AB Vista, Marlborough, UK; 5000 FTU/g) was supplemented in all the diets. Each basal diet was divided into two experimental diets: control diet (without enzyme supplementation), or supplemented with a combination of 0.2 g/kg mono-component protease and 0.1 g/kg of xylanase (XP). The β-(1-4)-endo-xylanase was from genetically modified *Trichoderma reesei*. The serine protease was produced by the sporulation-deficient *Bacillus licheniformis* strain. Birds receiving each of the two experimental diets were allocated equally into two groups gavaged with 1 mL distilled water or 1 mL mixed-species *Eimeria* oocysts solution on day 15. The mixed-species *Eimeria* spp. water-based solution containing 12,500 oocysts of *E. maxima*, 12,500 oocysts of *E. tenella*, and 62,500 oocysts of *E. acervuline* per 1 mL was prepared for a mild infection challenge. The ingredient composition of the basal diets is presented in Table 1 and the analyzed chemical composition of the experimental diets is presented in Table 2. The analyzed enzyme activities were, on average 311, 9500, and 18,700 units per kg for phytase, xylanase, and protease, respectively. The analyzed activities were 62%, 56%, and 124% of expected activities for phytase, xylanase, and protease, respectively.

### 2.2. Growth Performance, Intestinal Permeability, and Lesion Scoring

All animal experiment procedures were approved by the Institutional Animal Care and Use Committee at the University of Georgia, Athens, Georgia, USA (Protocol No: A2018-08-026). Birds and feed were weighed on d 0, 15, and 21. Mortality was monitored daily and used to calculate mortality-adjusted weight gain (WG), feed intake (FI), and gain: feed ratio. The intestinal permeability test was conducted on d 19, 5 d post infection (DPI). A 2.2 mg/mL fluorescein isothiocyanate dextran (FITC-d, MW 4000; Sigma-Aldrich, St. Louis, MO, USA) solution was prepared right before the test. One bird was randomly selected from each of the challenged group cages and orally gavaged with 1 mL FITC-d solution. Birds from unchallenged and no-enzyme treatment were also administrated with FITC-d as the control group. Blank blood sample from extra birds (provided with standard broiler feed) was collected to dilute FITC-d to prepare solutions for the standard curve. After 2 h of administration, birds were euthanized and blood samples were collected from the heart and pooled by treatment. Clotted blood was centrifuged at 1000× *g* for 12 min to separate the serum. The serum sample and standard solutions were measured by a spectrophotometer (Spectramax M5, Molecular Devices, San Jose, CA, USA) at an excitation wavelength of 485 nm and an emission wavelength of 528 nm. All blood samples were kept in darkness during the whole procedure. The serum FITC-d concentration is positively correlated to intestinal permeability. High blood FITC-d level indicates intestinal leakage due to gut breakage caused by *Eimeria* spp. invasion.

At the end of the study, a 0 to 4 (no lesion to severe lesion) scale grading was used to score the coccidia lesion severity in predefined intestinal regions [18]. The upper (duodenum), middle (jejunum and ileum), and ceca sections of the intestine were scored separately. Lesion scoring was carried out on three birds per cage.

### 2.3. Sample Collection

All the birds were euthanized by carbon dioxide asphyxiation on d 21. Jejunal digesta was collected from five birds per cage. The samples were oven-dried and ground for oligosaccharides composition analysis. Cecal contents were collected from two birds per cage and stored at dry ice for later SCFA analysis. Jejunal tissues were sampled from two birds per cage, snap-frozen in liquid N, and later stored at −80 °C before further analysis.

### 2.4. Quantitative Real-Time PCR Analysis

Quantitative real-time PCR was used to analyze gene expression related to tight junctions and intestinal nutrient transporters. Approximately 2 mm × 2 mm jejunal tissue was homogenized in QIAzol lysis reagent (QIAGEN, Hilden, Germany) and total RNA was extracted following the manufacturer’s instructions. Extracted RNA was converted to cDNA in a 96-well PCR system by the use of the High-Capacity cDNA Reverse Transcription Kit (Thermo Fisher Scientific, Waltham, MA, USA) after quantity measurement and dilution. Converted cDNA was diluted and the real-time PCR reaction was performed with reaction master mix iTaq Universal SYBR Green Supermix (Bio-Rad, Hercules, CA, U.S.) in a Step One Plus real-time PCR system (Thermo Fisher Scientific, Waltham, MA, U.S.). Samples were run in duplicate and the 2^(−ΔΔCt)^ method [19] was applied to analyze the results. All of the primers used in the experiments, including housekeeping and target genes, are listed in Table 3.

### 2.5. Chemical Analysis

All the diets were analyzed for chemical profiles using standard procedures. Samples were oven-dried at 100 °C for 24 h to determine the gravimetric difference and then dry matter was calculated (AOAC Method 934.01). Nitrogen content was measured using the combustion by nitrogen analyzer (AOAC Method 968.06). The Ankom 200 Fiber Analyzer was used to measure acid and neutral detergent fibers (Ankom Technology, Macedon, NY, USA). Minerals were measured by the Central Analytical Laboratory, University of Arkansas. Matrix-assisted laser desorption ionization mass spectrometry detection was used to analyze the oligosaccharides’ composition of the jejunal by the Complex Carbohydrate Research Center, University of Georgia, as previously described [17]. Gas chromatography (GC) was used to analyze the composition of cecal SCFA by a previously described method [20]. Briefly, 1 g cecal content sample was diluted in 3 mL deionized water and centrifuged at 10,000× *g* for 10 min. The supernatant was mixed well with 25% meta-phosphoric acid. After overnight freezing, the mixture was thawed and the supernatant was mixed with ethyl acetate at a ratio of 1:2. After vortexed and settled, the top layer of the mixture was transferred to a glass vial and analyzed on GC. 

### 2.6. Statistical Analysis

The data for Expt.1 and 2 were analyzed separately by ANOVA using the mixed model procedure of JMP Pro 14.1.0 (SAS Institute Inc., Cary, NC, USA). The two factors were the *Eimeria* challenge and enzymes supplementation. Main effects refer to when there are no significant interactions, whereas simple effects refer to when there are significant interactions of factors. In cases of a significant interaction effect being detected, Tukey was used to separate the significantly different means. The Kruskal–Wallis nonparametric statistical method was used to analyze intestinal lesion scores. Significance was declared at *p* ≤ 0.05.

## 3. Results

### 3.1. Growth Performance

In both Expt. 1 and 2, *Eimeria* challenge resulted in a significant reduction (*p* < 0.01) in WG, FI, and gain–feed ratio (Table 4 and Table 5). Supplemental XP increased (*p* < 0.05) FI in broilers fed LFLP diets (Expt. 2) but had no effect on growth performance of broiler fed HFAP diet (Expt. 1). In both experiments, there was no significant challenge × enzyme interaction for the growth performance responses.

### 3.2. Intestinal Permeability and Lesion Scores

**Figure 1:**
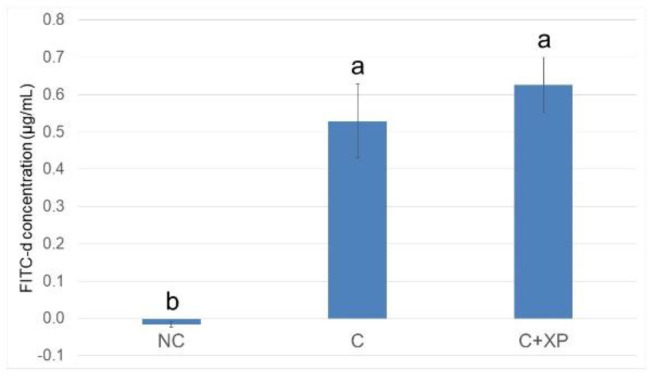
Fluorescein isothiocyanate dextran concentration (FITC-d, µg/mL) in serum of broiler chickens in response to feeding HFAP diet with or without enzyme supplementation (Expt. 1). Treatments with different letters are significantly different (*p* < 0.05). *N* = 6. NC, unchallenged (no enzyme) treatment; C, challenged (no enzyme) treatment; C+XP, challenged and supplemented with xylanase and protease treatment. The error bars represent the SEM values.

**Figure 2:**
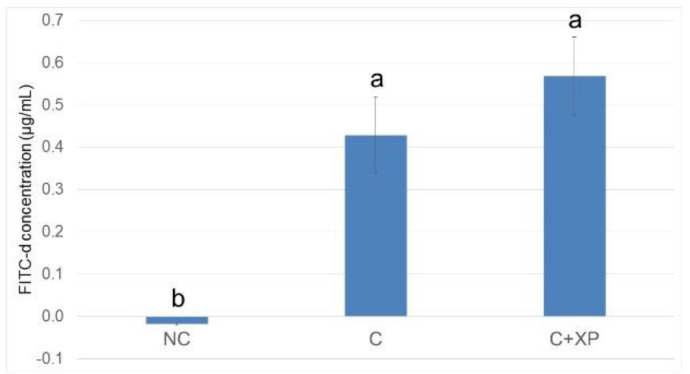
Fluorescein isothiocyanate dextran concentration (FITC-d, µg/mL) in serum of broiler chickens in response to feeding LFLP diet with or without enzyme supplementation (Expt. 2). Treatments with different letters are significantly different (*p* < 0.05). *N* = 6. NC, unchallenged (no enzyme) treatment; C, challenged (no enzyme) treatment; C+XP, challenged and supplemented with xylanase and protease treatment. The error bars represent the SEM values.

**Figure 3:**
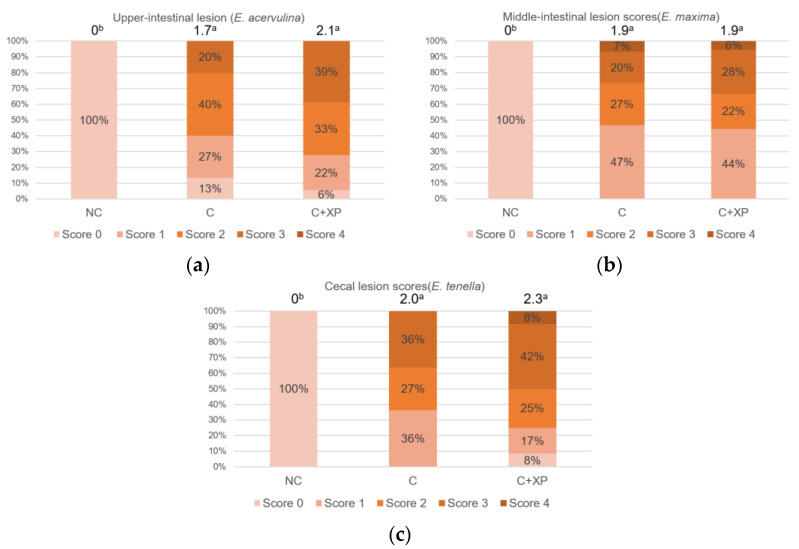
Lesion scores in the upper intestine, middle intestine, and ceca of broiler chicken in response to feeding HFAP diet with or without enzyme supplementation (Expt. 1; 6 days post-infection). Average scores for each treatment are presented at the top of the bar. Treatments with different letters are significantly different (*p* < 0.05): (**a**) Upper-intestine; (**b**) middle-intestine; (**c**) ceca. *N* = 6. NC, unchallenged—no enzyme treatment; C, challenged—no enzyme treatment; C+XP, challenged and supplemented with xylanase and protease treatment.

**Figure 4:**
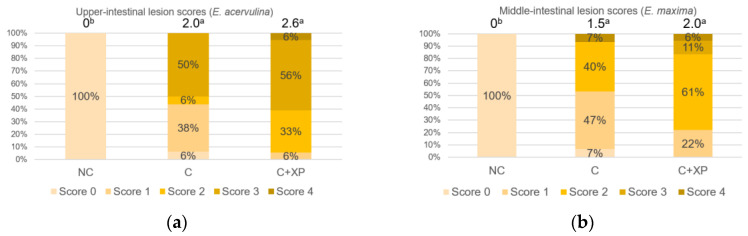

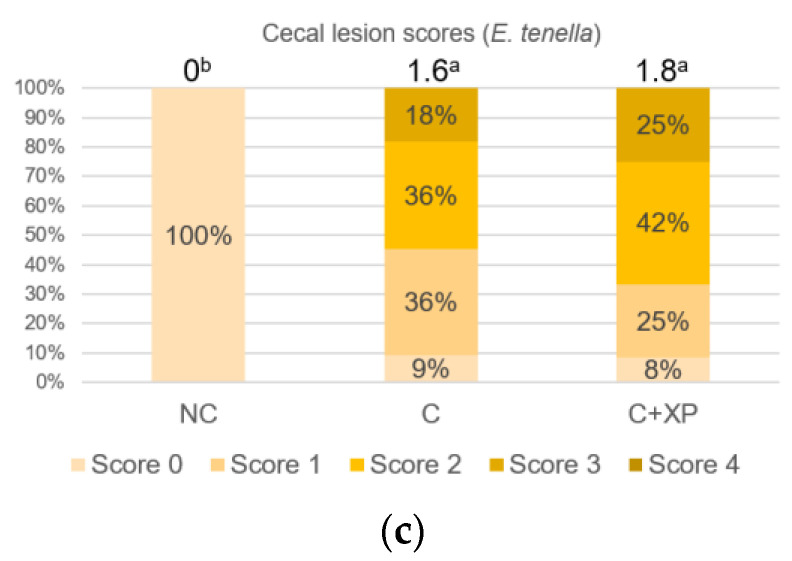
Lesion scores in the upper intestine, middle intestine, and ceca of broiler chicken in response to feeding LFLP diet with or without enzyme supplementation (Expt. 2; 6 days post-infection). Average scores for each treatment are presented at the top of the bar. Treatments with different letters are significantly different (*p* < 0.05): (**a**) Upper-intestine; (**b**) middle-intestine; (**c**) ceca. *N* = 6. NC, unchallenged—no enzyme treatment; C, challenged—no enzyme treatment; C+XP, challenged and supplemented with xylanase and protease treatment.

Figure 1 and Figure 2 show the gastrointestinal permeability response on day 19 (5 DPI). Enzyme supplementation had no effect on intestinal permeability compared with the control group (unchallenged treatment without enzymes) in both experiments. However, birds challenged with mixed *Eimeria* species showed higher (*p* < 0.01) serum FITC-d levels, indicating intestinal leakage due to gut breakage caused by *Eimeria* spp. invasion. The results of intestinal lesion scores are presented in Figure 3 and Figure 4, compared to the control group (unchallenged treatment without enzyme). *Eimeria* challenge resulted in higher (*p* < 0.01) lesion scores in the upper intestine, middle intestine, and ceca which were invaded by *E. acervulina*, *E. maxima*, and *E. tenella*, respectively, regardless of the type of basal diets. No significant effect on lesion score was observed for XP supplementation.

### 3.3. Gene Expression of Nutrients Transporters and Tight Junction Proteins

In Expt. 1, *Eimeria* challenge upregulated (*p* < 0.01) the expression of CLDN1, whereas enzyme supplementation downregulated (*p* < 0.01) the expression of the gene. Moreover, there was a significant (*p* < 0.01) enzyme × *Eimeria* challenge interaction for the expression of glucose transporters GLUT2 and GLUT5 (Table 6). In unchallenged groups, enzyme supplementation reduced (*p* < 0.05) the expression, while in *Eimeria*-challenged groups, the expression levels were unaffected. The highest and lowest GLUT2 and GLUT5 expressions were observed in unchallenged (without enzyme) and unchallenged (with enzyme) treatments, respectively. Downregulation (*p* < 0.05) of glucose transporter SGLT1 was observed in enzyme-supplemented diets in Expt. 1. In addition, the *Eimeria* challenge downregulated (*p* < 0.05) the expression of CAT2 and y+LAT1. Downregulation (*p* < 0.05) of amino acid transporter rBAT was observed in enzyme-supplemented diets in Expt. 1. The expression of JAM2 and PepT1 were not affected by the treatments in both experiments. In Expt. 2, there was no interaction between enzyme supplementation and *Eimeria* challenge on gene expression. In addition, *Eimeria* significantly upregulated (*p* < 0.01) tight junction protein CLDN1 and downregulated (*p* < 0.01) the expression of cationic amino acid transporters CAT2 and y+LAT1 whereas enzyme supplementation had no significant effect on the gene expression (Table 7).

### 3.4. Jejunal Oligosaccharides and Cecal Volatile Fatty Acids Profile

No significant main or interaction effects were observed on the profile of jejunal oligosaccharides in birds receiving the HFAP diet in Expt. 1 (Table 8). The profile of jejunal oligosaccharides in Expt. 2 (Table 9) indicates that birds challenged with mixed *Eimeria* spp. tended to have lower jejunal concentration of (Hex)3 (*p* = 0.062) and (Hex)5 (*p* = 0.059) but significantly lower (Hex)4 (*p* < 0.05), when fed LFLP diets. No significant enzyme effect nor interactions were observed. 

In both Expt. 1 and Expt. 2 (Table 10 and Table 11, respectively), there were significantly lower (*p* < 0.05) concentrations of SCFA acetate, butyrate, and total SCFA but higher (*p* < 0.05) concentrations of BCFA isobutyrate, and isovalerate in birds challenged with *Eimeria* spp. In birds receiving HFAP diets (Expt. 1), there was significant *Eimeria* × enzyme interaction for isobutyrate, isovalerate, and valerate (Table 10). The concentrations of BCFA were much greater (*p* < 0.05) in challenged birds than in unchallenged birds. Enzyme supplementation mollified *Eimeria*-induced increase in cecal BCFA isobutyrate and isovalerate contents. Lower (*p* < 0.05) cecal valerate was observed in birds fed without enzymes in challenged compared to unchallenged birds. However, when enzymes were supplemented, challenged birds had a higher (*p* < 0.05) cecal valerate value compared to unchallenged birds. No significant main enzymatic effects were found. For broiler chickens in Expt. 2, there was significant *Eimeria* × enzymes interaction (*p* < 0.05) for ceca content of acetate, butyrate, and total SCFA (Table 11). The concentrations of acetate, isobutyrate, and total VFA were highest (*p* < 0.05) in unchallenged–no enzyme treatments, but similar among the rest of the treatments.

## 4. Discussion

The objective of the two experiments reported herein was to investigate the potential and mechanisms of exogenous enzymes action on mitigating the negative effects of *Eimeria* challenge on growth performance, gene expression of tight junctions and nutrient transporters, and cecal fermentation pattern. *Eimeria*-induced reduction in broiler growth performance is positively correlated with infective dose [21,22]. Bodyweight gain of birds at 1 to 6 DPI (day 14 to day 20) can be linearly reduced from 27% to 49% when challenged by increasing oocysts doses of mixed *Eimeria* spp. (6250 *E. maxima*; 6250 *E.tenella*; 31,250 *E. acervuline* to 50,000 *E. maxima*; 50,000 *E. tenella*; 250,000 *E. acervulina*) [21]. A medium-low dose of *Eimeria* was used in the current study in order to develop a mild infection resulting in a 63% reduction in body weight gain at 6 DPI, which was more severe than expected but similar to what others have reported [23,24]. There are species differences in the extent to which *Eimeria* impacts growth performance. The less pathogenic species such as *E. praecox* and *E. mitis*, which do not cause lesions, generally have less influence on growth performance whereas *E. maxima*, which mainly invades jejunum and ileum, showed a more serious impairment on feed conversion ratio (FCR) [1,25]. Exogenous enzymes do not have anticoccidial activities. However, dietary inclusion of such enzymes may reduce the negative effect of coccidia infection via the independent effect of such enzymes on promoting growth performance, nutrient utilization, the integrity of intestinal epithelial cells, or bacterial balance. For example, enzyme complexes including xylanase or protease have been shown to significantly decrease FCR in broiler chickens challenged with coccidia [14,26,27]. In Expt. 2 of the current study, enzyme supplementation increased feed intake without any effect on WG or the gain–feed ratio. The enzyme’s effect during coccidia challenge is not universally observed. Enzyme supplementation had no significant mitigation effect on the impairment of the growth performance associated with coccidiosis in Parker et al. [28] or in Expt. 1 of the current study. Dietary and other factors may play a role in differences in response observed from different studies.

*Eimeria* challenge increases the permeability of the intestinal epithelium, and the extent of the damage can be assessed using fluorescein isothiocyanate-dextran (FITC-d). Higher levels of FITC-d in serum indicate greater FITC-d leakage from the intestine, revealing the severity of impairment of tight junction barriers [29]. Upon entering the host intestine, sporulated oocysts begin several cycles of asexual multiplication before sexual multiplication. During the asexual multiplication stages, a mass of merozoites is produced and penetrates the epithelial cells of the host, resulting in severe intestinal impairment and permeability defects [3]. In the current study, the levels of FITC-d detected in bird serum were greater in challenged birds compared with non-challenged birds. However, enzyme supplementation had no effect on gut leakage. The observation was likely because enzymes cannot directly target *Eimeria* oocyst nor reduce its multiplication, which is the primary cause of cell destruction and gut leakage. For the same reason, there was no lesion score reduction observed in enzyme-supplemented treatments.

On the other hand, observation in the current experiments on the expression of tight junction proteins indicates that the enzymes supplementation promoted intestinal integrity. Tight junctions are multi-functional protein complexes principally consisting of three families of transmembrane proteins: claudins, occludins, and junctional adhesion molecules. These complexes act as guards to seal the paracellular space between adjacent epithelial cells, regulating nutrient absorption and restricting pathogen entry. Claudin family proteins constitute the tight junction structural framework and each protein plays a specific function [30]. In the current study, in response to the mixed *Eimeria* challenge, the expression of claudin-1 at 6 DPI was significantly increased. The same change of claudin-1 was reported in previous coccidia studies [21,31]. The key inflammatory cytokine TNFα led to the increase in claudin-1 in IEC-18 cells, and increased expression of claudin-1 was observed in ulcerative colitis. It is speculated that the increased claudin-1 is related to inflammatory bowel disease [32,33,34]. When attacked by inflammatory pathogens, the intestinal paracellular structure, as well as osmotic balance, are damaged, requiring a mass replacement of barrier-forming proteins including claudin-1. This intensive replacement is clinically observed as diarrhea. This replacement can not only renew intestinal barriers but also protectively exclude invasive pathogens [34]. *Eimeria* spp. multiplies by invading and breaking down intestinal cells intercellularly and intracellularly. All of these *Eimeria*-induced activities stimulate the replacement of tight junctions, consequently increasing the expression of claudin-1, which was also observed in the current study. The data in the current study demonstrated the combined effect of xylanase and protease on decreasing the claudin-1 expression. It can be reasoned that the enzymes helped to partly alleviate *Eimeria*-induced intestinal barrier impairment and thus helped reduce the metabolic costs associated with the disease challenge. Similar findings were reported in animals receiving xylanase supplemented diet when challenged with Clostridium perfringens [35,36].

*Eimeria* infection causes the downregulation of nutrient transporters, which consequently contributes to possible depressed nutrient utilization and ultimately diminished growth performance [37]. In the current study, *Eimeria* infection significantly downregulated the expression of sugar transporters GLUT2 and GLUT5 in broilers fed HFAP diets (Expt. 1), although the decrease was rather nominal for birds receiving LFLP diets in Expt. 2. GLUT5, located on the apical side, is a facilitated-diffusion fructose transporter that facilitates the uptake of fructose into enterocytes. GLUT2, located at the basolateral side, takes over the sugar absorption transport once inside the cell and mediates the passive transport of glucose, fructose, and galactose out of enterocytes into the bloodstream [38,39]. Others have similarly observed the downregulation of intestinal GLUT2, GLUT5, and SGLT1 in *Eimeria*- and *Campylobacter jejuni*-challenged birds [7,22,37,40]. In the current study, amino acid transporters CAT2 and y+LAT1 were also downregulated in *Eimeria*-challenged groups regardless of the dietary fiber and protein levels. This observation is consistent with previous observations that *E. maxima* caused ileal and *E. tenella* caused jejunal CAT2/y+LAT1 downregulation [37]. CAT2 and y+LAT1 are basolateral amino acid transporters mediating the transport of cationic and neutral amino acids [41]. As important L-arginine transporters, the depression of CAT2 and y+LAT1 may contribute to the reported sharp drop of plasma arginine in *E. acervulina* challenged chickens [42].

The downregulation of nutrient transporter proteins caused by *Eimeria* challenge affects a wide range of sugar and amino acid transporter proteins such as GLUT2, GLUT5, SGLT1, PepT1, b0AT, b0+AT, EAAT3, rBAT, y+LAT2 [6,7,37,43]. The causes for the downregulation effect are not clear, but it is speculated that this response is a cell-mediated protective reaction in response to pathogenic invasion. By downregulating nutrient transporters in epithelial cells, a depleted nutritional environment is created to limit the development of parasites. In addition, the malnourished cells may trigger apoptosis and consequent epithelial renewal [6,37]. The interaction between *Eimeria* challenge and enzyme supplementation in HFAP diets (Expt. 1) showed that compared to the non-challenged treatments, enzyme supplementation upregulated the expression of GLUT2 and GLUT5 to possibly compensate for nutrient utilization deficit. The xylanase-mediated nutrient transporter upregulation was reported in broiler chickens challenged with *Clostridium perfringens* [35,44]. These observations suggest the potential of exogenous enzymes to alleviate some negative effects of *Eimeria* challenge in broiler chickens.

Exogenous enzymes or dietary fiber have been shown to influence the content and profile of oligosaccharides in the digestive tract [15,17,45,46]. Some of these oligosaccharides could serve prebiotic functions in birds. In a study by Lin and Olukosi [17], broiler chickens receiving diets with higher fiber content had a greater content of jejunal pentose-oligosaccharides as well as cecal SCFA. In the current study, the concentration of jejunal hexose-oligosaccharides was reduced in the challenged birds receiving LFLP diets (Expt. 2), which is likely due to the depressed feed intake in challenged birds. On the other hand, no significant difference was observed in the jejunal oligosaccharides content in the different HFAP treatments in Expt. 1. Interestingly, the *Eimeria* challenge increased the cecal concentration of BCFA which exclusively originates from protein fermentation, but decreased the concentration of acetate and butyrate which mainly originate from carbohydrates fermentation, regardless of the types of diet profile in the two experiments. The production of BCFA is considered a marker for estimating cecal protein fermentation [47], thus it can be speculated that *Eimeria* promoted cecal protein fermentation but inhibited the fermentation of carbohydrates. This is possibly due to a change in the proportion of protein and carbohydrates reaching the hindgut for fermentation or a shift in the microbial population inhabiting the hindgut of challenged, compared with non-challenged birds.

It is widely acknowledged that hindgut fermentation of carbohydrates is generally more beneficial than protein fermentation [48]. For example, butyrate resulting largely from carbohydrate fermentation is regarded as an energy source for enterocytes. Butyrate also plays a role in reducing inflammation and oxidative stress and in enhancing the colonic defense barrier. The supplementation of butyrate was reported to control coccidiosis [49]. The greater ceca BCFA content observed in *Eimeria*-challenged chickens may be explained by the observed downregulation in amino acid transporters. This may lead to impaired amino acids absorption in the small intestine of the host, and consequently leading to disproportionally large amounts of protein in the hindgut. Secondly, large amounts of epithelial cell debris produced by coccidiosis-induced intestinal damage ultimately flow to the ceca as potentially fermentable protein substrates. In addition, an *Eimeria*-induced cecal microflora shift has been indicated in previous studies [49], leading to an increase in Firmicutes (mucins and amino acids fermentation) and Proteobacteria (amino acids fermentation) and a decrease in Bacteroidetes (carbohydrates fermentation) [50,51,52]. Therefore, protein fermentation is promoted at the expense of carbohydrates fermentation. Instructively though, in the current study, enzyme supplementation mitigated the *Eimeria*-induced enhancement of cecal BCFA in HFAP diets (Expt. 1). These observations demonstrated a potential positive effect of enzyme supplementation in alleviating the shift in microbial fermentation patterns caused by *Eimeria* challenge.

## 5. Conclusions

In conclusion, *Eimeria* challenge triggered changes to the expression of claudin 1 and nutrient transporters irrespective of the diet types. In addition, *Eimeria* infection resulted in the promotion of cecal protein fermentation and inhibited carbohydrates fermentation. Exogenous enzymes showed the potential of alleviating *Eimeria*-induced intestinal gene expression changes and mitigating the unfavorable cecal fermentation pattern, and thus demonstrated that enzyme supplementation may benefit beyond nutrient improvement.

## Figures and Tables

**Table 1:** Ingredient compositions (g/kg) of the experimental basal diets.

Items	Expt. 1 (HFAP)	Expt. 2 (LFLP)
Corn	300	655
Wheat	200	0
Wheat bran	100	0
Soybean meal	310	260
Soybean oil	40	35
Titanium dioxide	5.0	5.0
Di-calcium phosphate	8.9	8.9
Limestone	15.6	15.6
Lysine	4.2	4.2
Methionine	3.4	3.4
Threonine	2.6	2.6
NaHCO_3_	2.0	2.0
Salt	3.5	3.5
Vitamin premix ^1^	2.5	2.5
Trace minerals premix ^2^	2.5	2.5
Phytase	0.1	0.1
Total	1000	1000
Calculated Nutrients and Energy, g/kg		
CP	211	181
ME, kcal/kg	2752	2988
Ca	9.5	9.2
Total P	6.0	5.0
Available P ^3^	2.8	2.7
Met	6.5	6.3
Cys	3.5	3.1
Met + Cys	10.0	9.4
Lys	14.4	12.7
His	5.5	4.9
Trp	2.6	2.0
Thr	10.3	9.4
Arg	14.0	11.7

^1^ Vitamin A, 3,527,360 IU/kg; vitamin D3, 1,399,921 ICU/kg; vitamin E, 19,400 IU/kg; vitamin B12, 8.8 mg/kg; menadione, 1102 mg/kg; riboflavin, 3527 mg/kg; d-pantothenic acid, 5467 mg/kg; thiamine, 970 mg/kg; niacin, 20,282 mg/kg; vitamin B6. 1455 mg/kg; folic acid, 573 mg/kg; biotin, 79 mg/kg. µg. ^2^ Calcium, 3.20–4.20%; manganese 13.40%; Zinc, 10.70%; magnesium, 2.68%; iron, 2.63%; copper, 40,000 ppm; iodine, 1000 ppm; selenium, 400 ppm. ^3^ Available P level included the matrix for the phytase. CP—crude protein; AP—adequate protein; LP—low protein; HF—high fiber; LF—low fiber.

**Table 2:** Analyzed composition (g/kg) of experimental diets.

Items	Expt. 1 (HFAP)	Expt. 2 (LFLP)
Dry matter	901	895
Crude protein	204	172
Calcium	8.66	10.21
Total Phosphorus	4.22	7.25
Acid detergent fiber	38.1	32.0
Neutral detergent fiber	138	88.0
(Hex)3	44.7	34.7
(Hex)4	58.8	38.2
(Hex)5	1.54	1.14
(Hex)6	0.14	0.34
(Pent)3	0.09	ND
(Pent)5	0.31	0.13

Hex—hexose; Pent—pentose; ND—not detected; AP—adequate protein; LP—low protein; HF—high fiber; LF—low fiber. (Pent)4 and (Pent)6 were not detected in diets.

**Table 3:** GenBank accession number, sequences of forward and reverse primers used for real-time PCR.

Gene Symbol	Accession Number	Full Name	Function	Forward Primer	Reverse Primer
18S	XR_003078042.1	18S ribosomal RNA	Housekeeping gene	AGCCTGCGGCTTAATTTGAC	CAACTAAGAACGGCCATGCA
Beta-actin	NM_205518.1	Beta-actin	Housekeeping gene	CAACACAGTGCTGTCTGGTGGTA	ATCGTACTCCTGCTTGCTGATCC
CLDN1	NM_001013611.2	Claudin-1	Tight junction	TGGAGGATGACCAGGTGAAGA	CGAGCCACTCTGTTGCCATA
JAM2	XM_025149444.1	Junctional adhesion molecule 2	Tight junction	AGCCTCAAATGGGATTGGATT	CATCAACTTGCATTCGCTTCA
PepT1 (SLC15A1)	KF366603.1	Peptide transporter-1	Peptide transporter	CCCCTGAGGAGGATCACTGTT	CAAAAGAGCAGCAGCAACGA
GLUT1 (SLC2A1)	NM_205209.1	Glucose transporter-1	Glucose transporter	CTTTGTCAACCGCTTTGG	CAGAATACAGGCCGATGAT
GLUT2 (SLC2A2)	XM_010716927.3	Glucose transporter-2	Glucose transporter	TCATTGTAGCTGAGCTGTT	CGAAGACAACGAACACATAC
GLUT5 (SLC2A5)	XR_005855627.1	Glucose transporter-5	Glucose transporter	TTGCTGGCTTTGGGTTGTG	GGAGGTTGAGGGCCAAAGTC
SGLT1 (SLC5A1)	NM_001293240.1	Sodium glucose transporter-1	Glucose transporter	GCCGTGGCCAGGGCTTA	CAATAACCTGATCTGTGCACCAGT
CAT2 (SLC7A2)	XR_005859133.1	Cationic amino acid transporter-2	Cationic amino acid transporter	TGCTCGCGTTCCCAAGA	GGCCCACAGTTCACCAACAG
y+LAT1 (SLC7A7)	XM_040665181.1	y+ L type amino acid transporter-1	Cationic amino acid transporter	CAGAAAACCTCAGAGCTCCCTTT	TGAGTACAGAGCCAGCGCAAT
rBAT (SLC3A1)	XM_040667709.1	Solute carrier family 3, member1	Dimerize with b^o,+^AT	CCCGCCGTTCAACAAGAG	AATTAAATCCATCGACTCCTTTGC

**Table 4:** Growth performance of the broiler chickens challenged or unchallenged with mixed *Eimeria* spp. in response to feeding diets with high fiber and adequate protein levels with or without enzymes supplementation (Expt. 1).

			Pre-Challenge Phase (d 0 to 15)			Challenge Phase (d 15 to 21)		
Basal	*Eimeria*	XP	WG, g	FI, g	Gain: Feed, g/kg	WG, g	FI, g	Gain: Feed, g/kg
HFAP	-	-	415	494	845	387	447	866
HFAP	-	+	404	511	791	400	453	882
HFAP	+	-	418	523	798	135	324	417
HFAP	+	+	407	510	797	127	312	407
Pooled SEM			13.90	18.01	18.84	15.2	11.0	33.4
Means for Main Effect of *Eimeria* challenge								
	-					393	450	874
	+					131	318	409
*p*-values						<0.001	<0.001	<0.001
Means for Main Effect of XP Supplementation								
		-	417	508	822	261	385	642
		+	406	510	794	263	383	641
*p*-values			0.427	0.924	0.170	0.550	0.957	0.397
Pooled SEM			9.83	12.73	13.33	10.76	7.78	23.58
*p*-values for interactions						0.448	0.287	0.535

*n* = 6 replicates for the simple effect; *n* = 12 replicates for the main effects. HF—high fiber; AP—adequate protein; XP—xylanase plus protease supplementation; WG—weight gain, g; FI—feed intake, g.

**Table 5:** Growth performance of the broiler chickens challenged or unchallenged with mixed *Eimeria* spp. in response to feeding diets with low fiber and low protein levels with or without enzymes supplementation (Expt. 2).

			Pre-Challenge Phase (d 0 to 15)			Challenge Phase (d 15 to 21)		
Basal	*Eimeria*	XP	WG, g	FI, g	Gain: Feed, g/kg	WG, g	FI, g	Gain: Feed, g/kg
LFLP	-	-	389	502	774	389	502	774
LFLP	-	+	390	507	769	384	454	845
LFLP	+	-	368	486	755	145	313	462
LFLP	+	+	423	557	759	145	324	447
Pooled SEM			40.5	54.6	43.95	28.91	21.86	58.45
Means for Main Effect of *Eimeria* Challenge								
	-					372	438	848
	+					145	318	455
*p*-values						<0.001	<0.001	<0.001
Means for Main Effect of XP Supplementation								
		-	379	494	764	252	367	657
		+	406	532	749	264	389	646
*p*-values			0.113	0.114	0.460	0.391	0.042	0.708
Pooled SEM			11.68	15.77	12.69	8.35	6.31	16.87
*p*-values for interactions						0.370	0.303	0.868

*n* = 6 replicates for the simple effect; *n* = 12 replicates for the main effects. LF—high fiber; LP—low protein; XP—xylanase plus protease supplementation; WG—weight gain, g; FI—feed intake, g.

**Table 6:** Effects of mixed *Eimeria* spp. infection and exogenous enzymes on gene expression of tight junctions and nutrient transporters in the jejunum of broiler chickens on 6 days post-infection (HFAP; Expt. 1).

Basal	*Eimeria*	XP	CLDN1	GLUT2	GLUT5	SGLT1	CAT2	y+LAT1	rBAT
HFAP	-	-	1.016	0.762 ^a^	1.108 ^a^	1.146	1.050	1.084	1.132
HFAP	-	+	0.411	0.072 ^b^	0.136 ^b^	0.237	0.846	0.975	0.538
HFAP	+	-	1.796	0.200 ^b^	0.305 ^b^	1.385	0.512	0.478	0.758
HFAP	+	+	1.070	0.183 ^b^	0.248 ^b^	0.892	0.503	0.394	0.395
Pooled SEM			0.172	0.067	0.120	0.477	0.187	0.150	0.277
Means for Main Effect of *Eimeria* Challenge									
	-		0.714	0.417	0.622	0.692	0.948	1.029	0.835
	+		1.433	0.191	0.277	1.139	0.507	0.436	0.577
*p*-values			0.002	0.010	0.014	0.155	0.039	0.002	0.171
Means for Main Effect of XP Supplementation									
		-	1.406	0.481	0.706	1.266	0.781	0.781	0.945
		+	0.740	0.127	0.192	0.564	0.674	0.684	0.467
*p*-values			0.004	<0.001	0.001	0.032	0.597	0.560	0.017
Pooled SEM			0.122	0.048	0.085	0.337	0.132	0.106	0.196
*p*-values for interactions			0.761	0.001	0.002	0.498	0.630	0.535	0.532

*n* = 6 replicates for the simple effect; *n* = 12 replicates for the main effects. HF—high fiber; AP—adequate protein; XP—xylanase plus protease supplementation. Means with different superscripts are significantly different (*p* < 0.05).

**Table 7:** Effects of mixed *Eimeria* spp. infection and exogenous enzymes on gene expression of tight junctions and nutrient transporters of jejunum in broiler chickens on 6 days post infection (LFLP; Expt. 2).

Basal	*Eimeria*	XP	CLDN1	GLUT2	GLUT5	SGLT1	CAT2	y+LAT1	rBAT
LFLP	-	-	1.207	1.110	1.659	7.454	1.070	1.074	5.438
LFLP	-	+	1.108	0.794	1.242	4.202	1.485	0.900	4.034
LFLP	+	-	3.577	0.635	1.099	4.277	0.494	0.482	3.835
LFLP	+	+	4.008	0.571	1.503	4.180	0.667	0.578	2.550
Pooled SEM			0.580	0.288	0.400	0.910	0.198	0.139	0.470
Means for Main Effect of *Eimeria* Challenge									
	-		1.158	0.952	1.451	5.828	1.277	0.987	4.736
	+		3.793	0.603	1.301	4.228	0.580	0.530	3.193
*p*-values			<0.001	0.292	0.731	0.143	0.002	0.006	0.129
Means for Main Effect of XP Supplementation									
		-	2.392	0.872	1.379	5.865	0.782	0.778	4.636
		+	2.558	0.683	1.373	4.191	1.076	0.739	3.292
*p*-values			0.784	0.562	0.988	0.126	0.155	0.796	0.183
Pooled SEM			0.410	0.204	0.283	0.643	0.140	0.098	0.333
*p*-values for interactions			0.661	0.700	0.351	0.148	0.549	0.373	0.952

*n* = 6 replicates for the simple effect; *n* = 12 replicates for the main effects. LF—high fiber; LP—low protein; XP—xylanase plus protease supplementation.

**Table 8:** Oligosaccharide profile (µg/mg) in jejunal digesta of broiler chickens challenged or unchallenged with mixed *Eimeria* spp. in response to feeding diets with high fiber and adequate protein levels with or without enzyme supplementation (Expt. 1).

Basal	*Eimeria*	XP	(Hex)3	(Hex)4	(Hex)5	(Hex)6	(Pent)3	(Pent)4	(Pent)5
HFAP	-	-	73	91	9.07	0.547	0.509	0.738	1.353
HFAP	-	+	13	180	8.38	ND	0.280	0.134	0.659
HFAP	+	-	60	78	4.81	0.838	0.983	0.586	1.001
HFAP	+	+	20	17	1.84	0.486	1.176	0.288	0.273
Pooled SEM			34	45	3.6	0.333	0.505	0.303	0.511
Means for Main Effect of *Eimeria* Challenge									
	-		102	136	8.73	0.274	0.395	0.436	1.006
	+		40	47	3.33	0.662	1.080	0.437	0.637
*p*-values			0.187	0.141	0.146	0.278	0.193	0.925	0.115
Means for Main Effect of XP Supplementation									
		-	67	85	6.94	0.692	0.746	0.662	1.177
		+	76	99	5.11	0.243	0.728	0.211	0.466
*p*-values			0.810	0.768	0.646	0.232	0.933	0.195	0.276
Pooled SEM			24.0	32.0	2.54	0.236	0.357	0.214	0.361
*p*-values for interactions			0.255	0.186	0.840	0.859	0.763	0.667	0.946

*n* = 6 replicates for diet effect; *n* = 12 replicates for the main effects. HF—high fiber; AP—adequate protein; XP—xylanase plus protease supplementation; Hex—hexose; Pent—pentose; ND—not detected.

**Table 9:** Oligosaccharide profile (µg/mg) in jejunal digesta of broiler chickens challenged or unchallenged with mixed *Eimeria* spp. in response to feeding diets with low fiber and protein levels with or without enzyme supplementation (Expt. 2).

Basal	*Eimeria*	XP	(Hex)3	(Hex)4	(Hex)5	(Hex)6	(Pent)3	(Pent)4	(Pent)5
LFLP	-	-	140	150	7.39	0.121	5.791	0.699	0.929
LFLP	-	+	134	154	6.27	ND	3.181	ND	1.158
LFLP	+	-	49	22	2.85	ND	2.109	0.333	0.350
LFLP	+	+	19	12	0.70	ND	2.257	0.501	0.448
Pooled SEM			49	51	2.84	0.064	2.258	0.348	0.633
Means for Main Effect of *Eimeria* Challenge									
	-		137	152	6.83	0.060	4.486	0.349	1.044
	+		34	17	1.78	ND	2.183	0.417	0.399
*p*-values			0.062	0.034	0.059	0.398	0.581	0.862	0.834
Means for Main Effect of XP Supplementation									
		-	95	86	5.12	0.060	3.950	0.516	0.639
		+	76	83	3.48	ND	2.719	0.250	0.803
*p*-values			0.765	0.974	0.676	0.375	0.644	0.490	0.103
Pooled SEM			35	36	2.01	0.045	1.596	0.246	0.447
*p*-values for interactions			0.804	0.894	0.840	0.372	0.595	0.274	0.933

*n* = 6 replicates for diet effect; *n* = 12 replicates for the main effects. LF—low fiber; LP—low protein; XP—xylanase plus protease supplementation; Hex—hexose; Pent—pentose; ND—not detected.

**Table 10:** Short-chain fatty acid profile (mM) in cecal content for the broiler chickens challenged or unchallenged with mixed *Eimeria* spp. in response to feeding diets with high fiber and adequate protein levels with or without enzyme supplementation (Expt. 1).

Basal	*Eimeria*	XP	Acetate	Propionate	Isobutyrate	Butyrate	Isovalerate	Valerate	Total VFA
HFAP	-	-	98	7.3	0.178 ^c^	23.6	0.601 ^b^	1.002 ^b^	131
HFAP	-	+	96	9.0	0.359 ^bc^	21.6	0.738 ^b^	1.199 ^b^	129
HFAP	+	-	75	14.3	1.758 ^a^	17.1	3.509 ^a^	1.649 ^a^	114
HFAP	+	+	63	11.2	0.968 ^b^	13.4	1.785 ^b^	1.117 ^b^	91
Pooled SEM			7.14	2.08	0.163	2.32	0.345	0.164	8.88
Means for Main Effect of *Eimeria* Challenge									
	-		97	8.2	0.303	23.8	0.721	1.126	132
	+		69	11.7	1.435	15.9	2.888	1.362	102
*p*-values			0.002	0.126	<0.001	0.013	<0.001	0.137	0.019
Means for Main Effect of XP Supplementation									
		-	87	10.8	0.968	20.3	2.055	1.325	122
		+	79	10.1	0.663	17.5	1.262	1.158	110
*p*-values			0.362	0.504	0.126	0.274	0.079	0.331	0.184
Pooled SEM			5.05	1.47	0.115	1.64	0.244	0.116	6.28
*p*-values for interactions			0.497	0.090	0.023	0.729	0.044	0.049	0.247

*n* = 6 replicates for diet effect; *n* = 12 replicates for main effects. HF—high fiber; AP—adequate protein; XP—xylanase plus protease supplementation. Means with different superscripts are significantly different (*p* < 0.05).

**Table 11:** Short-chain fatty acid profile (mM) in cecal content for the broiler chickens challenged or unchallenged with mixed *Eimeria* spp. in response to feeding diets with low fiber and protein levels with or without enzyme supplementation (Expt. 2).

Basal	*Eimeria*	XP	Acetate	Propionate	Isobutyrate	Butyrate	Isovalerate	Valerate	Total VFA
LFLP	-	-	116 ^a^	9.1	0.279	29.5 ^a^	0.664	1.373	157 ^a^
LFLP	-	+	87 ^b^	9.0	0.409	21.3 ^ab^	0.798	1.106	120 ^ab^
LFLP	+	-	68 ^b^	11.4	1.170	15.1 ^b^	2.313	1.251	99 ^b^
LFLP	+	+	71 ^b^	13.0	1.628	16.5 ^b^	3.674	1.645	108 ^b^
Pooled SEM			6.9	1.98	0.189	2.71	0.409	0.183	9.16
Means for Main Effect of *Eimeria* Challenge									
	-		103	8.8	0.264	25.8	0.621	1.220	140
	+		72	14.2	1.561	16.5	3.371	1.591	109
*p*-values			0.002	0.300	0.002	0.046	0.002	0.415	0.018
Means for Main Effect of XP Supplementation									
		-	92	10.2	0.724	22.3	1.488	1.312	128
		+	79	11.0	1.018	18.9	2.236	1.376	114
*p*-values			0.113	0.878	0.188	0.196	0.124	0.755	0.118
Pooled SEM			4.75	1.33	0.150	2.156	0.329	0.142	6.67
*p*-values for interactions			0.046	0.422	0.395	0.045	0.150	0.088	0.015

*n* = 6 replicates for diet effect; *n* = 12 replicates for main effects. LF—low fiber; LP—low protein; XP—xylanase plus protease supplementation. Means with different superscripts are significantly different (*p* < 0.05).
